# Beyond Motor Neuron Degeneration: Non-Motor Manifestations and Multisystem Involvement in Amyotrophic Lateral Sclerosis

**DOI:** 10.3390/jcm15218750

**Published:** 2026-11-09

**Authors:** Maria Veciunca, Medeea Roman, Vlăduț-Gabriel Vasiliu, Rodica Bălașa, Anca Moțățăianu

**Affiliations:** 1 Neurology Clinic, Colentina Clinical Hospital, 020125 Bucharest, Romania; maria.veciunca022025@rez.umfcd.ro; 2 Doctoral School, “George Emil Palade” University of Medicine, Pharmacy, Science, and Technology of Targu Mures, 540142 Targu Mures, Romania; 3 Emergency Institute for Cardiovascular Diseases “Prof. Dr. C.C. Iliescu”, 022322 Bucharest, Romania; vladut-gabriel.vasiliu022025@rez.umfcd.ro; 4 Department of Neurology, “George Emil Palade” University of Medicine, Pharmacy, Science, and Technology of Targu Mures, 540142 Targu Mures, Romania; rodica.balasa@umfst.ro (R.B.); anca.motataianu@umfst.ro (A.M.)

**Keywords:** amyotrophic lateral sclerosis, non-motor symptoms, multisystem involvement, dysautonomia, cognitive impairment, sleep disorders

## Abstract

Amyotrophic lateral sclerosis (ALS) is a fatal neurodegenerative disease that primarily affects the motor system through the degeneration of motor neurons in the motor cortex, brainstem nuclei, and anterior horn of the spinal cord. ALS typically has a focal onset characterized by muscle weakness, followed by progressive spreading to other body regions, with respiratory muscle failure representing the main life-limiting complication. Median survival is approximately 2–5 years after disease onset. In addition to the well-established motor manifestations, non-motor symptoms are increasingly recognized and may occur in approximately 50% of patients. These include behavioral and neuropsychiatric disturbances, sleep disorders, sensory abnormalities, autonomic dysfunction involving the cardiovascular, gastrointestinal, and genitourinary systems, as well as metabolic and endocrine alterations. Recent evidence supports the concept of ALS as a multisystem disorder characterized by substantial clinical, genetic, and neuropathological heterogeneity. Nevertheless, non-motor manifestations remain less extensively characterized than motor symptoms and are frequently underestimated in clinical practice. This narrative review summarizes the current evidence on non-motor manifestations in ALS, discusses their potential underlying mechanisms and impact on quality of life, and highlights areas requiring further investigation. A better understanding and systematic assessment of these manifestations may contribute to a more comprehensive and patient-centered approach to ALS care.

## 1. Introduction

Amyotrophic lateral sclerosis (ALS), also known as motor neuron disease (MND), is a neurodegenerative disease involving motor neurons in the motor cortex, brainstem and spinal cord [1,2], presenting with progressive motor impairment and often leading to death in 3 to 5 years due to respiratory failure [3,4,5].

Amyotrophic lateral sclerosis (ALS) represents a complex syndrome characterized by motor features such as limb and bulbar muscle weakness and fasciculations; however, a growing body of evidence demonstrates that non-motor symptoms (NMS) are part of the clinical picture of ALS and contribute significantly to a reduced quality of life [6]. These NMS include cognitive decline, neuropsychiatric features, sleep disorders, sensory disturbances, and autonomic dysfunction [7,8]. Recent studies have drawn attention to the NMS in ALS and it has been suggested that these non-motor symptoms may arise from pathological spread to other neighboring non-motor brain regions (frontal and temporal cortex, basal ganglia, and hypothalamus, a key integrator of the autonomic nervous system), a process also seen in other neurodegenerative diseases, such as Parkinson disease [8,9]. However, some of them may originate from muscle weakness, which makes the definition of non-motor symptoms difficult. Such symptoms are important for two reasons: (1) they have a significant impact on the patients’ quality of life, and (2) they can have a prediagnostic value as endophenotypes, as observed in other conditions in the field of neurodegenerative pathologies, such as Parkinson’s disease [10].

Given the limited availability of disease-modifying treatments, the management of these NMS is an important aspect of the management of patients with ALS. The full impact of non-motor symptoms in ALS is not yet fully understood, because attention has traditionally been focused on motor symptoms. This is also due to the possible underreporting of these symptoms by patients and their caregivers [1].

The term “non-motor manifestations” in ALS encompasses a heterogeneous group of symptoms with different underlying mechanisms. For the purposes of this review, we distinguish between manifestations potentially related to direct extramotor neurodegeneration, symptoms occurring secondarily to motor impairment, immobility, bulbar dysfunction, or respiratory muscle weakness, treatment-related manifestations, and symptoms attributable to comorbid conditions. Some manifestations may have mixed or incompletely understood mechanisms and cannot be assigned exclusively to a single category. Thus, symptoms such as dysphagia and sialorrhea may predominantly reflect bulbar motor dysfunction, pain may arise secondarily from immobility or musculoskeletal complications, and sleep-related hypoventilation primarily results from respiratory muscle weakness. Conversely, cognitive, neuropsychiatric, autonomic, sensory, and metabolic abnormalities may provide evidence of pathological involvement extending beyond the classical motor system. This operational distinction is applied throughout the review and is important when interpreting non-motor manifestations as evidence of multisystem involvement in ALS [9].

Although ALS has long been considered a disorder limited to the neuromotor system, accumulating evidence suggests that ALS is a multisystem disease, highlighting the relevance of non-motor features, which can significantly influence disease progression, quality of life, and survival outcomes [11]. From an immune system perspective, the collective dysregulation of immune cells and cytokines in ALS—correlating with higher disease burden, faster progression, and reduced survival—supports the concept of widespread systemic pro-inflammatory involvement and identifies potentially relevant therapeutic targets [12,13]. However, the hypothesis of ALS as a primary systemic disease remains debated. Although clinically relevant, systemic immune alterations do not necessarily indicate a systemic origin of pathology and may instead represent secondary or amplifying responses to motor neuron degeneration. Therefore, distinguishing between systemic involvement and systemic causation is essential when interpreting these findings.

Beyond immune-mediated mechanisms, emerging evidence also supports the involvement of neuroendocrine and metabolic regulatory autonomic centers in ALS. Metabolic and appetite dysregulation have emerged as consistent and clinically meaningful manifestations [14]. Recent studies have identified hypothalamic neuronal loss, proteinopathy, and structural volume reduction through both histological and neuroimaging approaches, suggesting that ALS pathology may extend to regions governing energy homeostasis and autonomic regulation. This hypothalamic involvement provides a potential mechanistic link between central neurodegeneration and systemic metabolic alterations observed in patients [15].

Given the growing literature on non-motor manifestations in ALS, the present review aims to extend beyond a descriptive catalogue of symptoms by providing an updated mechanistic and interpretative synthesis of the available evidence. In particular, we distinguish manifestations that may reflect intrinsic extramotor neurodegeneration from those occurring predominantly as secondary consequences of motor impairment, bulbar dysfunction, respiratory muscle weakness, treatment, or comorbid conditions. We further examine the strength and consistency of the available evidence, the potential prognostic relevance of individual manifestations, and areas in which current data remain conflicting or insufficient. By integrating clinical, neurophysiological, neuroimaging, pathological, and mechanistic evidence, this review seeks to provide a clinically meaningful framework for interpreting non-motor manifestations within the broader concept of ALS as a multisystem disorder.

## 2. Literature Search Strategy

This narrative review was based on a targeted literature search conducted primarily in PubMed between July 2024 and January 2025, with additional targeted updates during manuscript preparation. Search terms included combinations of “amyotrophic lateral sclerosis”, “ALS”, “non-motor symptoms”, and domain-specific terms related to cognitive and behavioral dysfunction, neuropsychiatric symptoms, autonomic dysfunction, sensory symptoms, metabolic and endocrine alterations, and sleep disorders. Additional searches were performed for relevant pathophysiological mechanisms.

The Parkinson’s Disease Non-Motor Symptoms Questionnaire (NMSQuest) [16] was used as a conceptual framework for organizing the ALS-related manifestations into six major domains: cognitive and behavioral dysfunction, neuropsychiatric manifestations, autonomic dysfunction, sensory disturbances, metabolic and endocrine alterations, and sleep-related disorders. Relevant peer-reviewed clinical studies, reviews, meta-analyses, mechanistic studies, and guidelines were considered. Recent publications were prioritized, while older seminal studies were retained when relevant. As this was a narrative review, no formal meta-analysis or risk-of-bias assessment was performed.

## 3. Non-Motor Symptoms in ALS

Non-motor symptoms are increasingly recognized as core components of ALS and may be related to the extracortical spread of the underlying pathology and may be associated with a poorer prognosis. Studying the spectrum of non-motor symptomatology is an important aspect of the management of patients with ALS, in terms of patient expectations, caregiver burden, disease progression and therapeutic options [17].

In a study involving 112 ALS patients, 25 non-motor symptoms were identified, of which the most common were: depression (47%), pain (26%), fatigue (24%), anxiety (18%), pseudobulbar affect (13%), and paresthesias (13%) [18].

In this narrative review, we have described in detail the non-motor symptoms that manifest in patients with ALS, highlighting their complexity and impact on quality of life. These symptoms have been divided into six main domains (Figure 1), each addressing a distinct area of patient functioning, such as: (a) cognitive and behavioral dysfunction, (b) neuropsychiatric manifestations, (c) autonomic dysfunction (dysfunctions of the cardiovascular, gastrointestinal and genitourinary systems), (d) sensory disturbances, (e) metabolic and endocrine alterations, and (f) sleep-related disorders. There are also other non-motor manifestations associated with the disease, which do not necessarily fall into the previous categories, but contribute to the complexity of the clinical picture seen in patients with ALS.

This article further provides an updated narrative overview of non-motor symptomatology in ALS based on clinical and paraclinical findings.

### 3.1. Cognitive and Behavioral Impairment/Dysfunction/Neuropsychological Impairment

Cognitive dysfunction is a key non-motor feature of ALS, affecting an estimated 30% to 75% of patients [19]. It can appear early in the disease course [20] and is associated with reduced survival [21], as well as a significant burden on caregivers.

Cognitive and behavioral changes in ALS are heterogeneous, spanning a continuum from subtle or subjective deficits to full frontotemporal dementia (FTD), collectively described as the ALS–frontotemporal spectrum disorder (ALS–FTSD) [22]. These impairments primarily involve executive function, attention, language, and verbal memory [23,24]. Overall, up to 50% of patients may experience some degree of cognitive or behavioral involvement [22], with prevalence increasing as the disease progresses.

Increasing evidence supports the view that ALS and FTD represent a single disease spectrum, ranging from predominantly motor to predominantly cognitive manifestations [25].

From a network-based perspective, ALS and frontotemporal lobar degeneration (FTLD), although clinically distinct, show overlapping patterns of structural and functional connectivity changes. Advanced MRI techniques—such as diffusion tensor imaging and resting-state fMRI—consistently demonstrate involvement of frontotemporal networks in both conditions, underpinning the cognitive, behavioral, and motor features seen across the ALS–FTLD continuum. Emerging MEG data, although still limited, further support this view by revealing altered dynamic interactions within these networks, reinforcing the concept of a network-based neurodegenerative spectrum [10].

Consensus criteria for the classification of cognitive and behavioral impairment in ALS are available within the ALS–frontotemporal spectrum disorder (ALS–FTSD) framework, including categories such as ALS with cognitive impairment (ALSci), ALS with behavioral impairment (ALSbi), combined cognitive and behavioral impairment (ALScbi), and ALS with frontotemporal dementia (ALS-FTD) [25]. In addition, ALS-specific screening instruments, particularly the Edinburgh Cognitive and Behavioural ALS Screen (ECAS), are widely used to identify cognitive and behavioral abnormalities in clinical practice [26]. Nevertheless, heterogeneity in assessment methods, normative data, and longitudinal trajectories remains an important consideration when comparing findings across studies.

Neurodegenerative diseases, such as ALS and FTD, are marked by the accumulation of misfolded proteins that disrupt cellular function and promote neurotoxicity. Under both conditions, RNA-binding proteins such as TDP-43 (TAR DNA-binding protein 43) and FUS (Fused in sarcoma) normally form dynamic, liquid-like condensates, but in diseases, they undergo abnormal phase transitions, mislocalize to the cytoplasm, and aggregate.

In particular, TDP-43 accumulation—characterized by ubiquitination and aggregation—serves as both a pathological hallmark and a potential driver of disease mechanisms [27,28]. Experimental evidence supports seeded aggregation and cell-to-cell propagation of pathological TDP-43, a mechanism sometimes described as ‘prion-like’; however, the extent to which this mechanism contributes to disease spread in human ALS remains uncertain. FUS-associated ALS represents a distinct proteinopathy and should not be assumed to follow the same propagation mechanism [29].

At the genetic level, these pathological processes are strongly linked to mutations in key ALS/FTD genes. Missense mutations in *TARDBP*, mainly affecting the aggregation-prone C-terminal domain, account for 3–5% of familial ALS cases and have also been associated with FTD [30]. Similarly, mutations in *FUS* promote cytoplasmic mislocalization and aggregation and are typically associated with severe, rapidly progressive ALS [31].

The hexanucleotide repeat expansion *C9ORF72*, the most common genetic cause of ALS and ALS-FTD, contributes to neurodegeneration through combined mechanisms, including loss of protein function, toxic RNA foci formation and accumulation of dipeptide repeat proteins [32,33]. Together, these genetic alterations highlight mechanistically distinct pathways in ALS–FTD that may converge on shared disturbances in RNA metabolism, proteostasis, and neuronal network function (Figure 2).

Regarding loneliness, several studies have investigated its link to mental health in ALS patients and their caregivers, showing a strong association with depression, anxiety, and even a desire to hasten death. However, its specific impact on cognitive and behavioral function, as well as cortical networks, remains unclear in ALS [34].

Although eye movement abnormalities are not typically considered a core feature, they may serve as useful markers of neurodegeneration in ALS, particularly when evaluated alongside cognitive and behavioral changes. Bedside assessment of these abnormalities is a quick and specific method for detecting cognitive impairment [35]. Overall, ALS is characterized by a rapid decline, affecting both motor and cognitive function [36].

Although cognitive and behavioral deficits in ALS are well described, their onset and progression relative to motor symptoms remain insufficiently understood. Cross-sectional studies suggest that cognitive impairment worsens with disease progression, particularly in domains commonly affected in ALS, such as language, executive function, and verbal fluency. However, longitudinal data are mixed: about 62% of studies report stable cognition over 6–24 months, while 31% describe decline in a subset of patients—often associated with executive dysfunction and shorter disease duration at baseline. Interpretation of these findings is limited by small sample sizes and short follow-up periods in many studies [37,38,39].

The pathophysiology of cognitive dysfunction is likely multifactorial; among contributing factors, hypoventilation due to respiratory impairment may play a role [40].

### 3.2. Neuropsychiatric Symptoms

Some studies suggest that neuropsychiatric symptoms may precede the onset of motor manifestations in ALS [41]. These symptoms (Figure 3) are among the most extensively studied and include: apathy (loss of interest), anxiety, depression, suicidal ideation, fatigue, and pseudobulbar affect [10,42]. Approximately one-third of patients develop depression, while the prevalence of anxiety remains less clearly defined [43].

Among neuropsychiatric features in ALS, apathy has been the most extensively studied. Its presence is associated with poorer quality of life, faster disease progression, and reduced treatment adherence, and recent multicenter data suggest that behavioral disturbances may worsen over time [17].

Depression, with an estimated prevalence of 25–40%, is a serious yet often underrecognized comorbidity in ALS [44,45]. Evidence indicates a bidirectional relationship: pre-existing depression may increase the risk of developing ALS [41], while patients with ALS are themselves at higher risk of depression [43]. In some cases, depressive symptoms may even precede motor onset, although their exact impact on disease progression and quality of life remains unclear. The mechanisms underlying depression in ALS are still poorly understood. There is a lack of dedicated neuroimaging and post-mortem data, and no studies have clearly defined its pathogenic link to the neurodegenerative process. By analogy with other neurodegenerative diseases, potential contributors may include prefrontal cortical thinning, disruption of mood-related networks, neurotransmitter imbalance, and neuroendocrine changes, though these remain speculative. Overall, the molecular basis of depression in ALS is insufficiently characterized, highlighting the need for multidisciplinary clinicopathological research to clarify its underlying mechanism [46,47,48]. Fatigue affects approximately 44–83% of patients with ALS and is likely multifactorial. Contributing factors include depression, reduced vital capacity, poor nutritional status, nocturia, sleep disturbances, muscle cramps and weakness, overall functional decline, and medication side effects, including Riluzole [49,50].

Pseudobulbar affect occurs in 20–50% of patients, particularly in those with bulbar onset [51]. It is characterized by sudden, involuntary episodes of laughing or crying that are disproportionate or inappropriate to the context, often with minimal provocation. These symptoms can significantly impair social interactions and overall quality of life [50]. More broadly, psychological, existential and social support factors play a major role in shaping quality of life in ALS patients [52].

Neuropsychiatric symptoms in ALS are closely linked to cognitive and behavioral changes following diagnosis [53]. Among these, dyspnea—a common feature of ALS— is strongly associated with anxiety, depression, and reduced quality of life. Understanding the interplay between dyspnea, anxiety, depression and quality of life is essential for effective management [54,55]. Longitudinal data further support this relationship: a prospective study of 36 patients highlighted the close association between motor disability and psycho-emotional disturbances such as anxiety and depression [56]. Notably, dyspnea appears to impact depression and quality of life largely through indirect pathways, particularly via fatigue and anxiety. Recognizing these mechanisms can help clinicians target interventions more effectively [54]. Overall, these findings underscore the need for broader symptom management strategies in ALS, beyond noninvasive ventilation alone [57].

To better understand the mechanisms underlying neuropsychiatric manifestations, ALS should no longer be considered as a pathology confined to motor neurons, but rather as a network-based neurodegenerative disease marked by progressive circuit dysfunction. Early cortical hyperexcitability, along with impaired inhibitory GABAergic interneuron activity, disrupts intracortical balance and facilitates pathological spread across interconnected neural systems [58]. While initially described in the motor cortex, similar alterations are increasingly recognized in the associative and limbic networks [59].

Degeneration and disintegration of cortico-limbic circuits—particularly prefrontal–amygdala, prefrontal–striatal, and hippocampal–cingulate pathways—support a neurobiological substrate for the neuropsychiatric features of ALS. Reduced prefrontal inhibitory control over limbic structures may contribute to emotional lability and impaired affect regulation, while fronto-striatal involvement is linked to apathy, executive dysfunction, and behavioral disinhibition. In parallel, the transsynaptic spread of TDP-43 pathology across cortical and subcortical regions supports a model of progressive network involvement, extending pathology beyond motor systems into domains of cognition, motivation, and socioemotional processing [59,60]. Overall, these findings support the view of ALS as a multisystem network disorder, with cortico-limbic degeneration playing a central role in shaping its neuropsychiatric profile.

Turning to serotonergic neurotransmission, growing evidence suggests that serotonergic neurotransmission is also altered in ALS. Early studies reported degeneration of brainstem serotonergic neurons and reduced projections to the hippocampus, indicating that serotonergic dysfunction is part of a broader network-level dysfunction.

Importantly, serotonin (5-HT) appears to influence both motor and non-motor aspects of the disease. Disrupted 5-HT signaling may contribute to cortical hyperexcitability and impaired regulation of glutamatergic circuits, further linking neurotransmitter imbalance to ALS pathophysiology [61].

### 3.3. Autonomic Dysfunction

ALS is increasingly recognized as a multisystem disorder that extends beyond the motor system to involve the autonomic nervous system. Despite this, dysautonomia is often overlooked in clinical practice. It is estimated to affect about one-third of patients, most commonly presenting with gastrointestinal and urinary symptoms. In most cases, dysfunction is mild (85%), with moderate forms in a smaller proportion and severe generalized dysautonomia being rare. Greater impairment has been reported in patients with upper motor neuron involvement [62] (Figure 4).

Autonomic symptoms can occur at any stage of the disease. They are often subclinical early, but in advanced stages may manifest as severe episodes, sometimes described as “autonomic storms” [63].

Notably, dysautonomia may contribute to sudden death in some ALS patients, even when nocturnal pulse oximetry is normal [64]. Importantly, when identified, certain autonomic symptoms can be managed, leading to meaningful improvements in quality of life [63].

**Figure 4:**
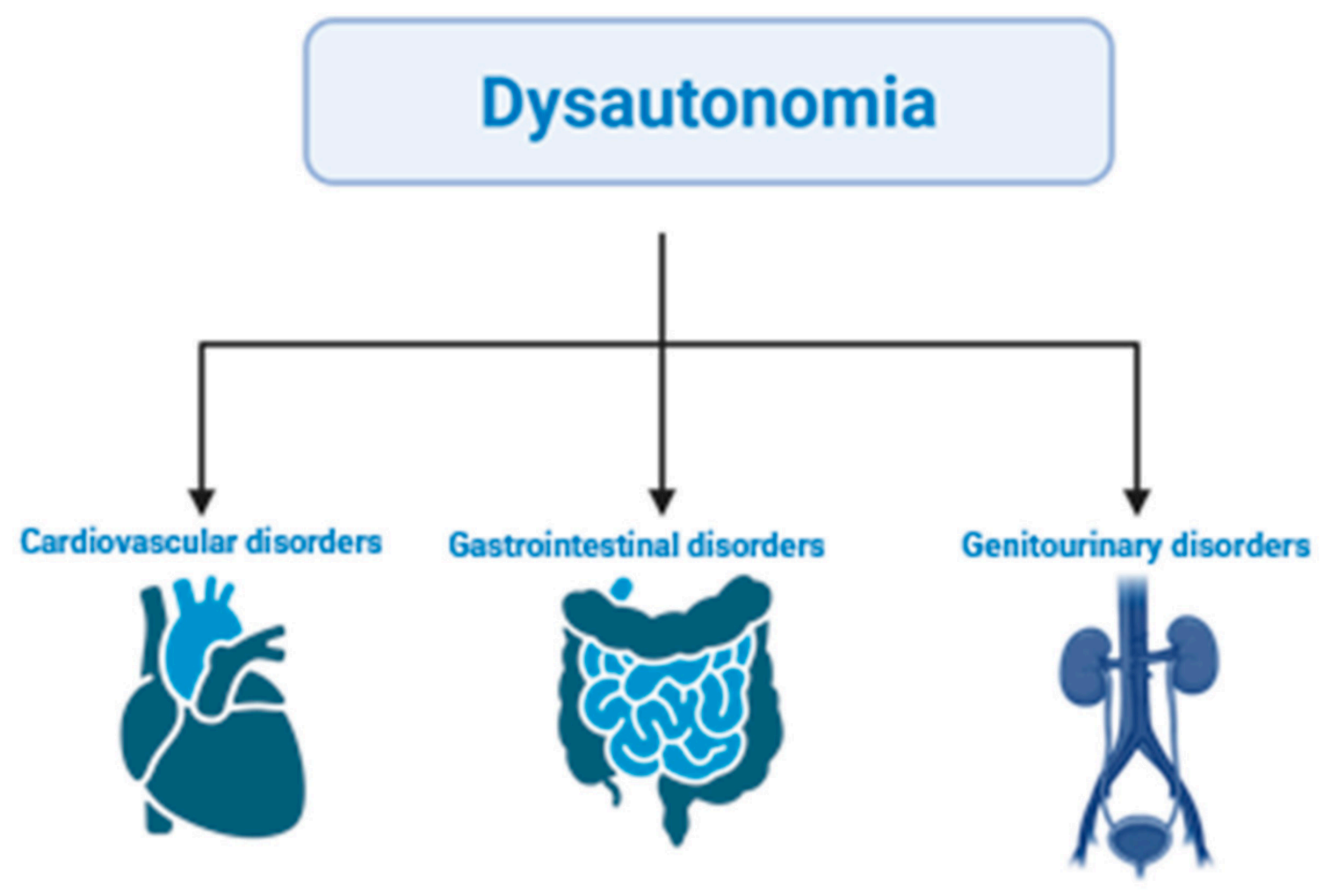
Dysautonomia in amyotrophic lateral sclerosis (ALS), including cardiovascular, gastrointestinal, and genitourinary autonomic dysfunction. ALS, amyotrophic lateral sclerosis.

#### 3.3.1. Cardiovascular Autonomic Dysfunction

Cardiovascular and cardiac autonomic dysfunction in ALS patients can be detected even in the early stages of the disease [65,66].

Heart rate variability (HRV), a key marker of autonomic dysfunction, reflects the balance between sympathetic and parasympathetic activity. Low-frequency (LF) components are influenced by both systems, while high-frequency (HF) components reflect parasympathetic activity. Spectral analysis of HRV in ALS patients shows reduced spectral power in both LF and HF bands, along with an increased LF/HF ratio, indicating an imbalance in autonomic control. In addition, reduced baroreceptor sensitivity has been reported [67,68] (Figure 5).

Further studies using standard autonomic testing and HRV analysis demonstrate decreased total and HF power-particularly in the supine position- suggesting impaired parasympathetic (vagal) control of cardiac function [69]. These alterations are more pronounced in patients with bulbar involvement. Overall, the findings point to early, often subclinical, autonomic dysfunction in ALS, with predominant impairment of parasympathetic cardiac regulation (Figure 5).

Prolonged QTc intervals and increased QTc dispersion -markers of impaired sympathetic activity-have been observed in terminally ill ALS patients on unassisted ventilation. These changes may contribute to an increased risk of sudden death and are thought to reflect severe degeneration of intermediolateral neurons in the upper thoracic spinal cord [70].

Orthostatic hypotension, while common in other neurodegenerative disorders such as Parkinson’s or Alzheimer’s disease, appears to be relatively rare in ALS [65,71,72].

In advanced stages, particularly in mechanically ventilated patients, episodes of “autonomic storm” have been reported, characterized by paroxysmal hypertension, sinus tachycardia and sudden drops in blood pressure [73,74].

Elevated plasma noradrenaline levels (an indirect marker of sympathetic hyperactivity) have been reported in sporadic ALS, likely reflecting secondary effects of respiratory failure and lower motor neuron dysfunction rather than a primary feature of disease [75].

Muscle sympathetic nerve activity (MSNA), a direct measure of sympathetic control over vascular resistance, shows a different pattern: it is increased in early ALS but tends to decline as the disease progresses and disability worsens [68,76,77].

In addition, high-resolution ultrasound (HRUS) studies have identified vagus nerve atrophy–particularly in patients with bulbar ALS, suggesting structural involvement of autonomic pathways [78].

Recent cardiac magnetic resonance (CMR) studies in ALS patients without prior heart disease have identified both structural changes- reduced ventricular volumes and masses- and functional impairment, including lower ejection volumes, compared to healthy controls [79].

Similarly, 123I-MIBG cardiac scintigraphy has shown increased cardiac sympathetic activity in ALS, a pattern associated with risks such as sudden cardiac death and stress-induced cardiomyopathy [80].

Overall, cardiovascular dysautonomia can significantly impact quality of life and survival in ALS, yet it is not routinely assessed. Evidence suggests that cardiovagal dysfunction is more common in patients with bulbar onset, who tend to have higher resting heart rates and blood pressure. Interestingly, these abnormalities do not correlate with disease duration or clinical severity, raising the possibility that autonomic dysfunction may occur early and in parallel with motor neuron degeneration, rather than as a purely secondary phenomenon [63].

#### 3.3.2. Gastrointestinal Manifestations and Autonomic Involvement

Gastrointestinal and bulbar-related manifestations in ALS include dysphagia, sialorrhea, nausea, abdominal pain, constipation, and fecal incontinence [65,81]. However, these manifestations do not share a single pathophysiological mechanism. Dysphagia should be regarded primarily as a manifestation of bulbar motor dysfunction rather than as evidence of autonomic involvement. Similarly, sialorrhea in ALS is predominantly caused by impaired clearance of saliva resulting from swallowing dysfunction, orofacial weakness, and reduced spontaneous swallowing, rather than by excessive salivary production [82,83,84,85]. Although alterations in salivary or autonomic regulation have also been reported [86], these findings should be distinguished from the substantially more common motor mechanism underlying sialorrhea. Dysphagia and sialorrhea are therefore discussed in this section because of their close interaction with gastrointestinal function, nutrition, and aspiration risk, but they should not be interpreted as primary evidence of gastrointestinal autonomic neurodegeneration. Clinically, sialorrhea can be highly distressing, leading to skin maceration, worsening dysarthria, sleep disturbances, and increased risk of aspiration pneumonia. It may also interfere with non-invasive ventilation adaptation and contribute to fatigue, embarrassment, social withdrawal, and reduced quality of life [87,88,89,90].

Sialorrhea in ALS should be evaluated by trained clinicians using standardized tools such as the OSS scale and ALSFRS-R, as early recognition may improve quality of life and help guide prognosis [91]. Despite its clinical relevance, data on the relationship between sialorrhea, ALS phenotype, and facial muscle weakness remain limited, highlighting the need for further research. Sialorrhea increases both physical and psychosocial burden for patients and their caregivers, contributing to reduced quality of life. Compared with Parkinson’s disease, this symptom remains relatively underrecognized in ALS and needs greater clinical attention [84]. Dysphagia assessment can be performed using fiberoptic endoscopic evaluation of swallowing (FEES), one of the gold-standard methods. This technique allows detailed evaluation of swallowing physiology, laryngeal sensitivity, and aspiration risk, while guiding dietary modifications and rehabilitation strategies. Based on these findings, appropriate liquid viscosity and food consistency can be recommended. Therapeutic approaches, including electrical stimulation and targeted swallowing exercises, have shown benefit [92,93]. Importantly, early detection and management of dysphagia are associated with improved outcomes and quality of life in ALS patients [94,95]. By contrast, evidence more directly supporting gastrointestinal autonomic or enteric involvement comes from abnormalities of gastrointestinal motility. Gastrointestinal motility is coordinated by the autonomic and enteric nervous systems (ENS), including Auerbach’s myenteric plexus and Meissner’s submucosal plexus [96]. In sporadic ALS, delayed gastric emptying and prolonged colonic transit have been reported. Importantly, these abnormalities were not associated with bulbar involvement, immobility, or disease stage [97], suggesting that, at least in a subset of patients, gastrointestinal dysfunction may involve mechanisms beyond secondary consequences of motor impairment. Experimental studies in TDP-43 transgenic models have demonstrated intestinal dysfunction and abnormalities involving the myenteric plexus, supporting the biological plausibility of enteric nervous system involvement in ALS [98]. However, the extent and clinical significance of ENS pathology in human ALS remain uncertain (Figure 6).

Gastrointestinal motor dysfunction in ALS may occur even in the absence of overt symptoms. Noninvasive assessments have demonstrated delayed gastric emptying and prolonged colonic transit times in these patients, suggesting subclinical involvement of gastrointestinal motility [97].

Constipation is relatively common in ALS and should be regarded as a multifactorial manifestation rather than as unequivocal evidence of autonomic dysfunction. Potential contributors include reduced mobility, altered nutritional and fluid intake, medication effects, and autonomic or enteric nervous system dysfunction [65]. In contrast, fecal incontinence is rare. Although single-fiber EMG studies show abnormalities of the external anal sphincter in ALS, residual muscle contraction is usually sufficient to maintain continence [99,100].

#### 3.3.3. Genitourinary Autonomic Dysfunction

Sexuality in patients with ALS has received limited attention in research, despite its clear impact on the quality of life. Available studies suggest that decreased libido, increased passivity in both partners, and reduced intimacy are common challenges. These changes are largely attributed to progressive physical weakness and altered body image, which can undermine confidence and strain relationships [101]. Sexual dysfunction may also have an autonomic component, in addition to reduced mobility and diminished sexual desire [62].

Regarding the female reproductive system, epidemiological studies show a slightly lower prevalence of ALS in women, suggesting a possible protective role of female hormones. Some studies also report that menopause before age 50 is associated with earlier ALS diagnosis [102].

Overall, sexuality remains an important yet often overlooked issue for many ALS patients and their partners. This topic is rarely discussed in clinical practice, highlighting the need for better information, counseling and multidisciplinary support to address sexual health and improve quality of life in ALS [101].

Urinary symptoms are relatively common in ALS, with lower urinary tract symptoms reported in 4–45% of patients [65]. These manifestations are often related to neurogenic bladder (Figure 7), with urinary incontinence being the most common complaint. Many patients experience both storage and voiding difficulties, commonly due to detrusor-sphincter dyssynergia and detrusor overactivity, often combined with incomplete sphincter relaxation [81].

Post-void residual volumes greater than 50 mL are observed in about 24–35% of patients and are associated with motor and lower limb impairment. Urinary retention is also frequent, particularly in patients with prominent spasticity, and may warrant urological evaluation [81,103]. Interestingly, lower urinary tract symptoms appear independent of age, disability or disease progression, while female sex appears to offer some protective effects. Early urinary symptoms have also been associated with reduced survival [104,105]. Although symptom severity is generally moderate, lower urinary tract symptoms may have a meaningful impact on quality of life and warrant clinical attention, particularly because potentially treatable abnormalities such as urinary retention and neurogenic bladder dysfunction have been described [104,105].

From a neuroanatomical perspective, dysautonomia in ALS reflects degeneration of both central and spinal autonomic structures. Loss of preganglionic sympathetic neurons in the thoracic intermediolateral column, along with parasympathetic neuron degeneration in the sacral cord, contributes to cardiovascular, gastrointestinal, and genitourinary dysfunction.

In addition, neuronal loss in brainstem nuclei—such as the dorsal motor nucleus of the vagus, nucleus ambiguus, and medullary raphe—disrupts parasympathetic regulation, while hypothalamic involvement further impairs homeostatic control. Together, these findings support the view that dysautonomia in ALS arises from widespread involvement of central and spinal autonomic networks, supporting the multisystem nature of the disease [106,107].

The pathophysiological mechanisms underlying dysautonomia in ALS likely involve both central and peripheral processes [65]. Degeneration of intermediolateral neurons and brainstem autonomic nuclei is compounded by oxidative stress and neuroinflammation, while excitotoxicity further disrupts autonomic circuits [107]. In addition, alterations in the gut–brain axis and impaired vagal signaling may promote systemic inflammation, further affecting central autonomic networks [108,109]. Together, these mechanisms help explain the cardiovascular, gastrointestinal, and genitourinary manifestations of dysautonomia observed in ALS [65].

### 3.4. Sensory Disturbances

Although ALS is primarily considered a motor disorder, sensory symptoms are frequently reported by patients [110]. Pain may precede the onset of overt motor symptoms in a subset of patients [111]. Muscle cramps, although common in ALS and potentially painful, are primarily related to motor-system dysfunction and should not be interpreted as evidence of primary sensory-system involvement.

According to the International Association for the Study of Pain, pain is defined as “an unpleasant sensory and emotional experience associated with, or resembling that associated with, actual or potential tissue damage” [36]. In ALS, pain is common but highly variable, with reported prevalence ranging from 15 to 85%. Recent studies indicate that approximately half to two-thirds of patients experience pain, with overall prevalence of about 60% [112,113,114].

Pain in ALS may arise through different mechanisms. Pain potentially related to direct sensory-system involvement includes neuropathic pain, whereas secondary nociceptive pain may result from muscle cramps, spasticity, immobility, postural abnormalities, or musculoskeletal complications. Accordingly, secondary pain related to motor dysfunction or immobility should be distinguished from pain potentially reflecting direct sensory-system involvement and should not, by itself, be considered evidence of multisystem neurodegeneration. Neurophysiological studies have demonstrated abnormalities of somatosensory evoked potentials in subsets of patients with ALS, supporting the possibility of involvement of ascending sensory pathways in addition to motor-system degeneration [115].

Pain significantly affects quality of life for both patients and caregivers and often adds to the burden of an already rapidly progressive disease [36,113,116]. Some studies suggest pain becomes more frequent with disease progression, while others report no clear relationship between pain and disease stage [117]. Overall, the natural history of pain in ALS remains poorly understood, largely because of limited longitudinal data [118].

Despite its prevalence and impact, pain in ALS remains underrecognized and undertreated. Mechanisms are diverse, and effective treatments are limited [111]. Although the World Health Organization highlights pain control as a core component of palliative care, there is currently no evidence-based pharmacological treatment for pain management in ALS [119]. Although ALS and sensory neuropathies are traditionally viewed as distinct conditions, some authors suggest they may overlap within a broader disease spectrum [120]. Sensory symptoms–most commonly tingling and superficial and deep hypoesthesia–typically follow a distal-to-proximal pattern and are more frequently reported in the lower limbs [121].

Electrophysiological studies often demonstrate a progressive sensory–motor axonal neuropathy, supporting the concept of a parallel neurodegenerative axonopathy within the ALS spectrum [122]. In addition, reduced or absent motor responses in distal muscles are characteristic findings, reflecting degeneration of anterior horn cells in the spinal cord [110].

In conclusion, although amyotrophic lateral sclerosis has traditionally been considered a purely motor neuron disorder, increasing evidence indicates that pain processing is frequently altered in ALS. Beyond secondary nociceptive causes, both peripheral and central mechanisms appear to contribute to pain perception. Neurophysiological and quantitative sensory testing studies have identified abnormalities consistent with small-fiber dysfunction and altered nociceptive thresholds, while experimental data point to impaired descending inhibitory control and possible central sensitization. Neuroimaging findings further show involvement of extramotor cortical and subcortical regions related to the affective and cognitive dimensions of pain. Together, these findings support the concept that pain in ALS is a multidimensional phenomenon with complex neurobiological mechanisms, rather than simply a consequence of motor degeneration [111,123,124].

From a diagnostic perspective, ALS with sensory symptoms must be carefully distinguished from ALS mimics. Importantly, even in spinal ALS, mild sensory neuropathy may be present, particularly in neurophysiological studies and biopsy findings [121].

### 3.5. Metabolic and Endocrine Alterations

This section addresses key metabolic and endocrine changes in ALS, including hypermetabolism and energy imbalance, alterations in carbohydrate and lipid metabolism, endocrine dysregulation, clinical phenotype, nutritional status and their impact on survival.

#### 3.5.1. Hypermetabolism and Energy Imbalance

ALS is increasingly recognized as a disorder associated with systemic energy imbalance. Many patients develop hypermetabolism, characterized by increased resting energy expenditure disproportionate to muscle mass and reduced physical activity. This metabolic shift often precedes advanced motor decline and contributes to progressive weight loss and reduced body mass index (BMI) [125]. Importantly, hypermetabolism is not exclusively a consequence of muscle atrophy. It likely reflects intrinsic alterations in mitochondrial function, neuroinflammation, and hypothalamic regulation of energy homeostasis [14]. Persistent negative energy balance has been consistently linked to faster functional decline and reduced survival in ALS [126,127].

Metabolic dysfunction in ALS extends beyond overall energy imbalance to include disturbances in carbohydrate and lipid metabolism. Several studies report impaired glucose tolerance and insulin resistance, indicating altered peripheral glucose handling. At the same time, increased lipid utilization and changes in circulating lipid profiles have been observed.

Interestingly, hyperlipidemia and higher premorbid BMI have been associated with improved survival in some cohorts, suggesting that greater lipid availability may help offset increased energy demands. Together, these findings support the concept of systemic metabolic reprogramming in ALS, rather than a purely catabolic state secondary to motor neuron degeneration [128].

#### 3.5.2. Endocrine Axis Dysregulation

Endocrine pathways involved in appetite regulation, metabolism and neuroprotection are also altered in ALS. Changes in circulating levels of leptin, ghrelin, insulin, insulin-like growth factor 1 (IGF-1), and glucagon-like peptide 1 (GLP-1) suggest dysfunction of the hypothalamic-peripheral endocrine axis [129].

Reduced leptin levels often reflect decreased fat mass, while altered ghrelin signaling may represent compensatory responses to negative energy balance. Insulin resistance and disrupted IGF-1 signaling may affect both metabolic control and neuronal survival. GLP-1, known for its metabolic and potentially neuroprotective effects, has also emerged as a molecule of interest in ALS-related metabolic dysfunction.

Together, these hormonal changes support the concept of impaired systemic metabolic regulation with potential implications for disease progression [129,130,131].

Metabolic alterations in ALS appear to influence disease progression rather than simply reflect secondary changes. Lower BMI at diagnosis, rapid weight loss, and hypermetabolism have been consistently associated with poorer outcomes. In contrast, higher baseline BMI and certain lipid abnormalities have been linked to longer survival in several studies.

These findings suggest that the metabolic phenotype interacts with clinical presentation, progression rate, and overall prognosis. Therefore, metabolic and endocrine disturbances should be considered key non-motor features of ALS, with important mechanistic and prognostic implications [14,132].

### 3.6. Sleep-Related Disorders

Sleep–wake disturbances are common in neurodegenerative diseases, including ALS. However, not all sleep-related complaints should be interpreted as primary non-motor manifestations. Sleep disruption may occur secondarily to motor impairment through immobility, muscle cramps, fasciculations, and difficulties with repositioning, while other disturbances may reflect respiratory dysfunction or, less clearly, alterations in central sleep–wake regulation. Muscle cramps and fasciculations should therefore be regarded primarily as motor-related contributors to impaired sleep rather than as evidence of extramotor neurodegeneration [133].

Respiratory dysfunction is another major contributor. Nocturnal hypoventilation and obstructive sleep apnea (OSA) are among the most frequent causes of sleep disturbance in ALS [134]. Sleep-related hypoventilation mainly results from phrenic nerve degeneration and diaphragmatic weakness, leading to carbon dioxide retention during REM sleep. As the disease progresses, hypercapnia extends to non-REM sleep and eventually to daytime, marking chronic hypercapnic respiratory failure. Sleep-related hypoventilation should therefore be regarded primarily as a secondary consequence of respiratory motor impairment rather than as direct evidence of non-motor neurodegeneration, although other sleep disturbances may also involve central sleep–wake and autonomic regulatory networks. Common symptoms include fragmented sleep, daytime sleepiness, morning headaches, and dyspnea.

OSA appears more prevalent in men and in patients with spinal-onset ALS, while it may be less common in bulbar-onset disease, where tongue atrophy reduces pharyngeal collapse [135]. The presence of OSA before ventilatory support has been associated with shorter survival [134], although it often reflects underlying respiratory muscle weakness that primarily drives disease progression.

Parasomnias are abnormal behaviors or movements occurring during sleep or at sleep–wake transitions, typically arising from partial arousals in non-REM or REM sleep. REM sleep behavior disorder (RBD) is characterized by persistent muscle activity during REM sleep, leading to dream-enactment behaviors such as movements or vocalizations, which may result in injury or falls. RBD is most commonly associated with neurodegenerative diseases, particularly synucleinopathies [136,137].

Beyond secondary respiratory mechanisms, hypothalamic and brainstem involvement may also contribute to sleep–wake disturbances in ALS. Neurodegeneration affecting these regulatory networks may interfere with circadian and sleep architecture, although the extent to which these abnormalities represent intrinsic extramotor pathology remains uncertain. Hypothalamic involvement has been increasingly recognized in ALS and may provide a mechanistic link between neurodegeneration, metabolic regulation, and sleep–wake dysfunction [15].

## 4. Discussion

The growing recognition of non-motor manifestations in ALS supports a broader view of the disease as a multisystem neurodegenerative disorder; however, the interpretation of these manifestations requires caution. Not all symptoms traditionally grouped under the term “non-motor” necessarily reflect intrinsic extramotor neurodegeneration. A clinically meaningful distinction should therefore be made between manifestations that may result from direct involvement of extramotor neural systems and those arising predominantly as secondary consequences of motor impairment, bulbar dysfunction, respiratory muscle weakness, treatment effects, or comorbid conditions. This distinction is particularly relevant when interpreting non-motor symptoms as evidence of multisystem involvement and represents an important framework for integrating the heterogeneous findings discussed in this review.

Among the domains reviewed, cognitive and behavioral abnormalities appear to have the most established association with intrinsic extramotor ALS pathology. Their relationship with the ALS–frontotemporal spectrum, together with converging neuroimaging, neuropathological, genetic, and molecular evidence involving frontotemporal networks and TDP-43 pathology, provides relatively strong support for direct disease-related involvement beyond the motor system [22,27,29]. Autonomic dysfunction also appears to represent genuine multisystem involvement in a subset of patients, although the evidence is more heterogeneous and may be influenced by disease stage, respiratory impairment, and treatment [62,65,106]. Sensory abnormalities and pain provide additional evidence of involvement beyond the classical motor system, but their interpretation is complicated by the coexistence of secondary musculoskeletal and immobility-related mechanisms [36,121,124]. Metabolic and endocrine abnormalities, including hypermetabolism, weight loss, and hypothalamic involvement, are increasingly supported by clinical and mechanistic studies, although the extent to which these changes represent primary ALS pathology rather than downstream systemic consequences remains incompletely resolved [14,15,126].

In contrast, several manifestations frequently grouped under the broad category of non-motor symptoms are more appropriately interpreted as secondary or mixed phenomena. Dysphagia is primarily a consequence of bulbar motor dysfunction, while sialorrhea largely results from impaired salivary clearance caused by swallowing and orofacial weakness rather than from excessive salivary production [82,83,86,87]. Similarly, pain related to immobility, postural abnormalities, or musculoskeletal complications should be distinguished from pain associated with direct sensory-system involvement [36,111,112]. Sleep-related hypoventilation is predominantly explained by respiratory muscle and diaphragmatic weakness and should therefore not be considered, in isolation, evidence of extramotor neurodegeneration [133,134]. Gastrointestinal complaints such as constipation may also result from multiple interacting factors, including reduced mobility, decreased fluid intake, nutritional changes, medication effects, and autonomic dysfunction [65,97]. These examples illustrate why a simple binary classification of symptoms as “motor” or “non-motor” may be insufficient and why their underlying mechanism should be considered when interpreting multisystem involvement in ALS.

The prognostic significance of non-motor manifestations also varies considerably across domains. Cognitive and behavioral impairment has been associated with reduced survival [21], while cardiovascular autonomic dysfunction, particularly reduced heart rate variability, may identify patients at increased risk of adverse outcomes [64]. Metabolic abnormalities appear to have particularly relevant prognostic implications, as hypermetabolism, rapid weight loss, and lower body mass index have been consistently associated with faster functional decline or shorter survival [126,127,132]. In contrast, the prognostic significance of several other manifestations, including pain, depressive symptoms, gastrointestinal complaints, and urinary dysfunction, remains less consistent or insufficiently characterized. Sleep-related respiratory abnormalities may also be associated with poorer outcomes [134]; however, their prognostic significance is closely intertwined with the severity of respiratory muscle dysfunction. Therefore, the presence of a non-motor manifestation should not automatically be interpreted as an independent prognostic marker, and its underlying mechanism and relationship with overall disease severity should be considered.

Several limitations of the current evidence should be acknowledged. Much of the literature on non-motor manifestations in ALS is based on relatively small cohorts, cross-sectional designs, heterogeneous definitions, and variable assessment tools, which limits direct comparison across studies. In addition, the contribution of disease stage, respiratory dysfunction, bulbar involvement, treatment effects, and comorbidities is not always adequately controlled, making it difficult to determine whether individual manifestations reflect intrinsic extramotor pathology or secondary consequences of advanced disease. Longitudinal studies using standardized ALS-specific assessment tools, integrated with neuroimaging, neurophysiological, autonomic, metabolic, and biomarker data, are needed to better define temporal relationships, mechanistic pathways, and prognostic relevance. Such studies may also help identify clinically meaningful non-motor phenotypes and improve patient stratification in future therapeutic research [9,10].

## 5. Conclusions

Amyotrophic lateral sclerosis (ALS) remains a complex and multifactorial neurodegenerative disorder with a profound impact on quality of life and survival. Despite advances in understanding its genetic background, the underlying molecular mechanisms and prognostic factors remain only partially understood, and therapeutic options are still limited. Non-motor symptoms represent an important yet often underrecognized dimension of ALS, contributing significantly to disease burden and clinical variability. Better characterization and systematic assessment of these manifestations—distinct from motor symptoms—are essential to clarify their role in disease progression and prognosis. Incorporating non-motor symptom evaluation into routine clinical practice, using standardized tools, may improve patient-centered care and support the development of targeted interventions. Ultimately, a comprehensive multidisciplinary and palliative approach remains essential for prolonging survival and optimizing quality of life in patients with ALS [138].

## Figures and Tables

**Figure 1:**
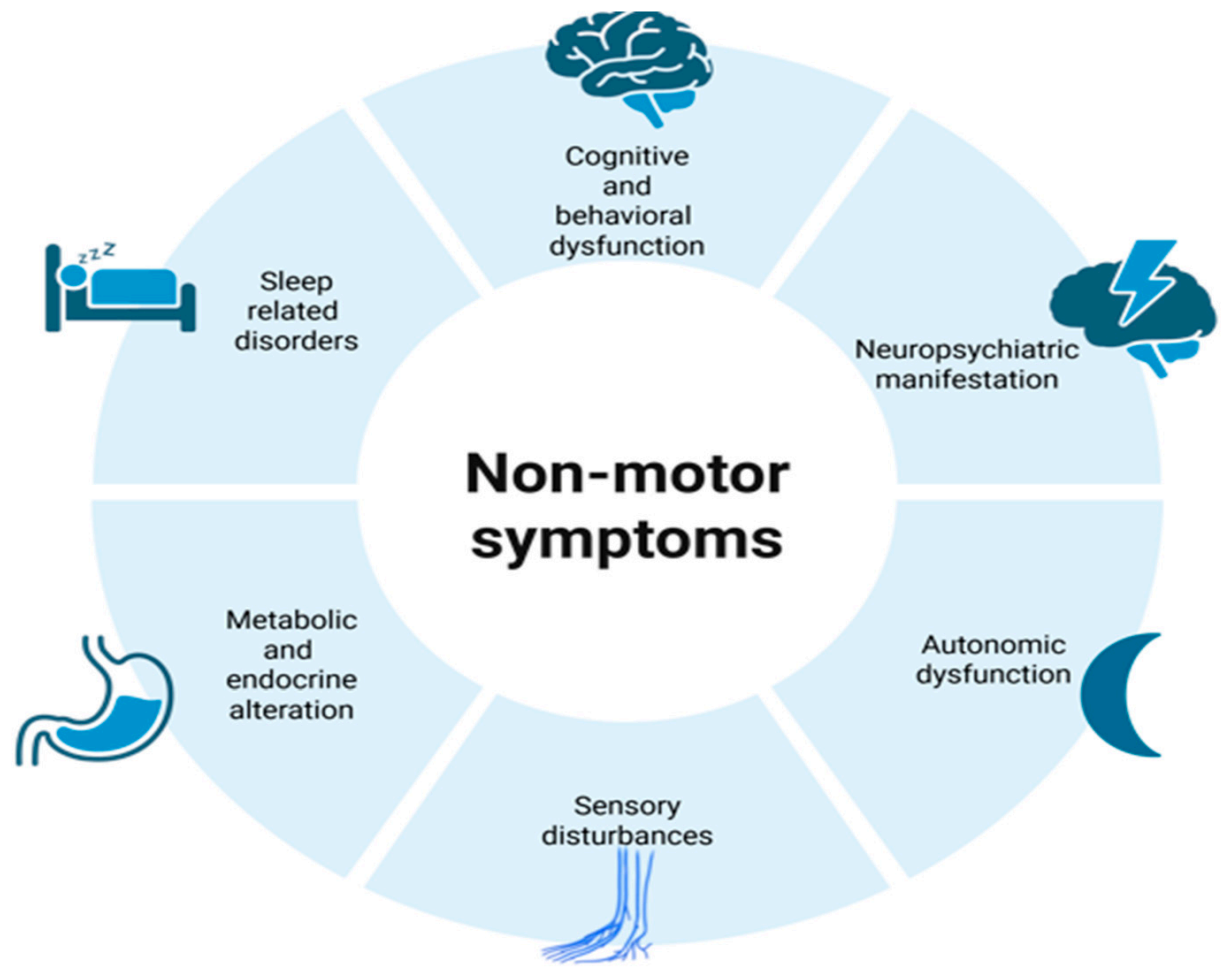
The main non-motor symptoms in ALS, divided into 6 areas: cognitive and behavioral dysfunction, neuropsychiatric manifestations, autonomic dysfunction, sensory disturbances, metabolic and endocrine alterations and sleep-related disorders. ALS—amyotrophic lateral sclerosis.

**Figure 2:**
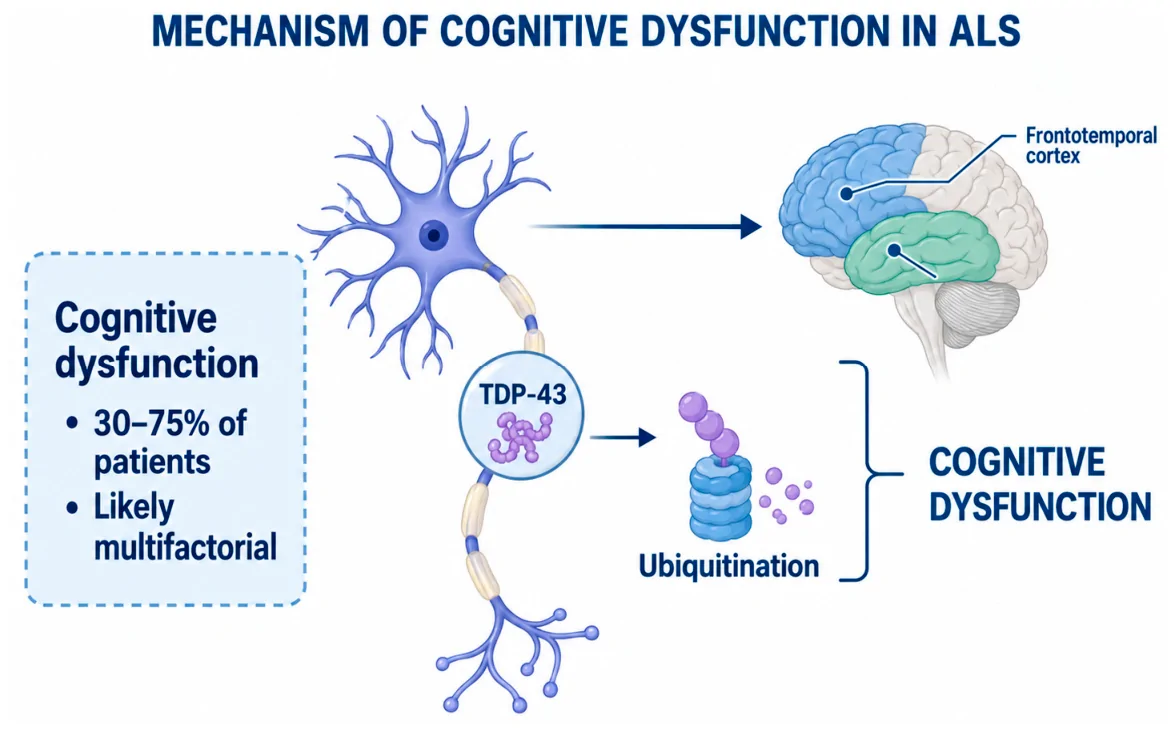
Proposed mechanisms of cognitive dysfunction in amyotrophic lateral sclerosis (ALS). Cognitive dysfunction, reported in approximately 30–75% of patients, is likely multifactorial and may involve frontotemporal cortical changes together with TDP-43 proteinopathy, including aggregation and ubiquitination. ALS, amyotrophic lateral sclerosis; TDP-43, TAR DNA-binding protein 43. Illustration generated using artificial intelligence for visualization purposes.

**Figure 3:**
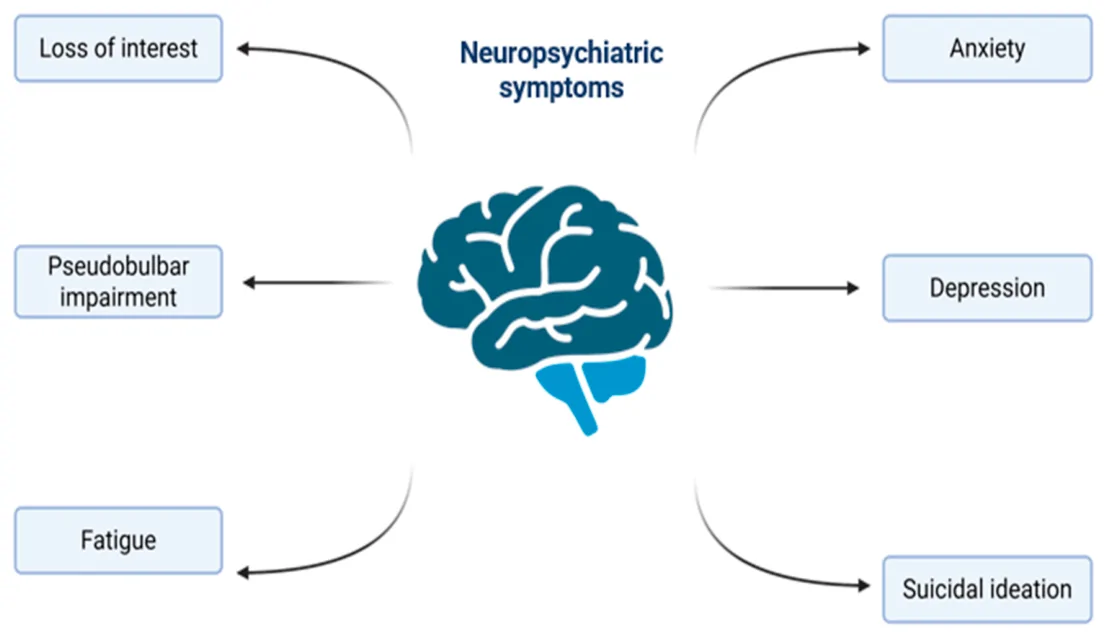
Neuropsychiatric symptoms in ALS.

**Figure 5:**
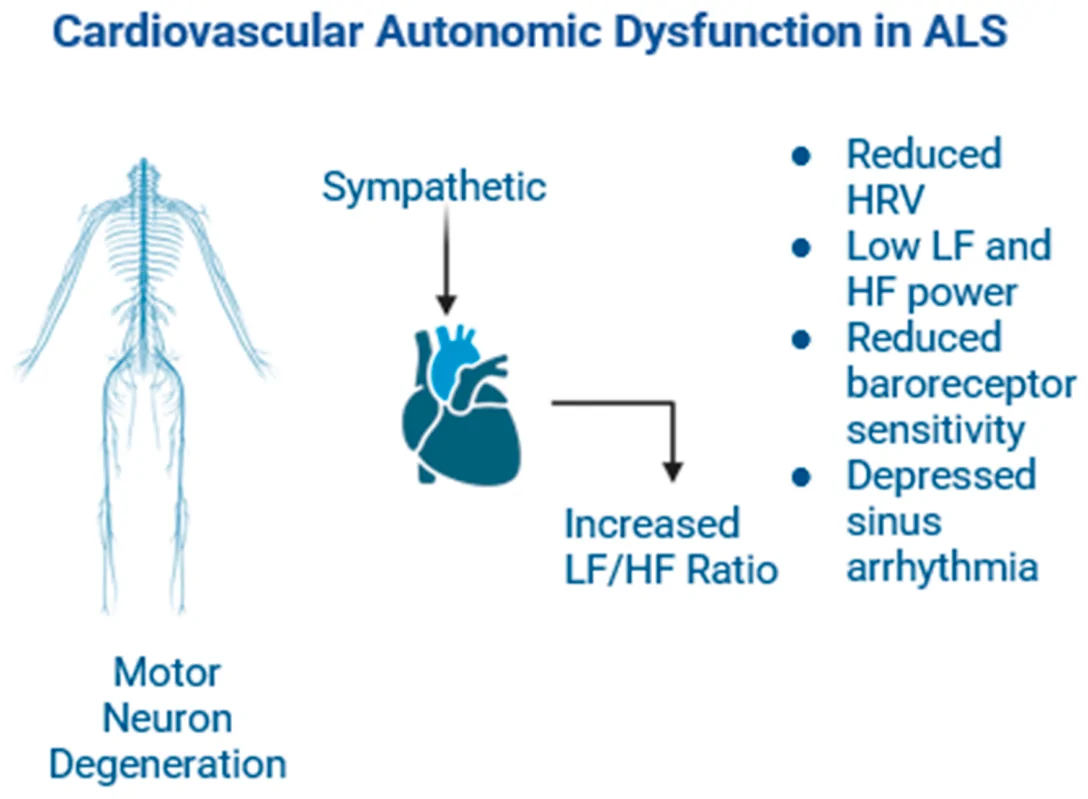
Cardiovascular autonomic dysfunction in amyotrophic lateral sclerosis (ALS). Heart rate variability abnormalities may include reduced low-frequency (LF) and high-frequency (HF) power, an increased LF/HF ratio, reduced baroreflex sensitivity, and impaired parasympathetic cardiac regulation. HRV, heart rate variability; LF, low frequency; HF, high frequency.

**Figure 6:**
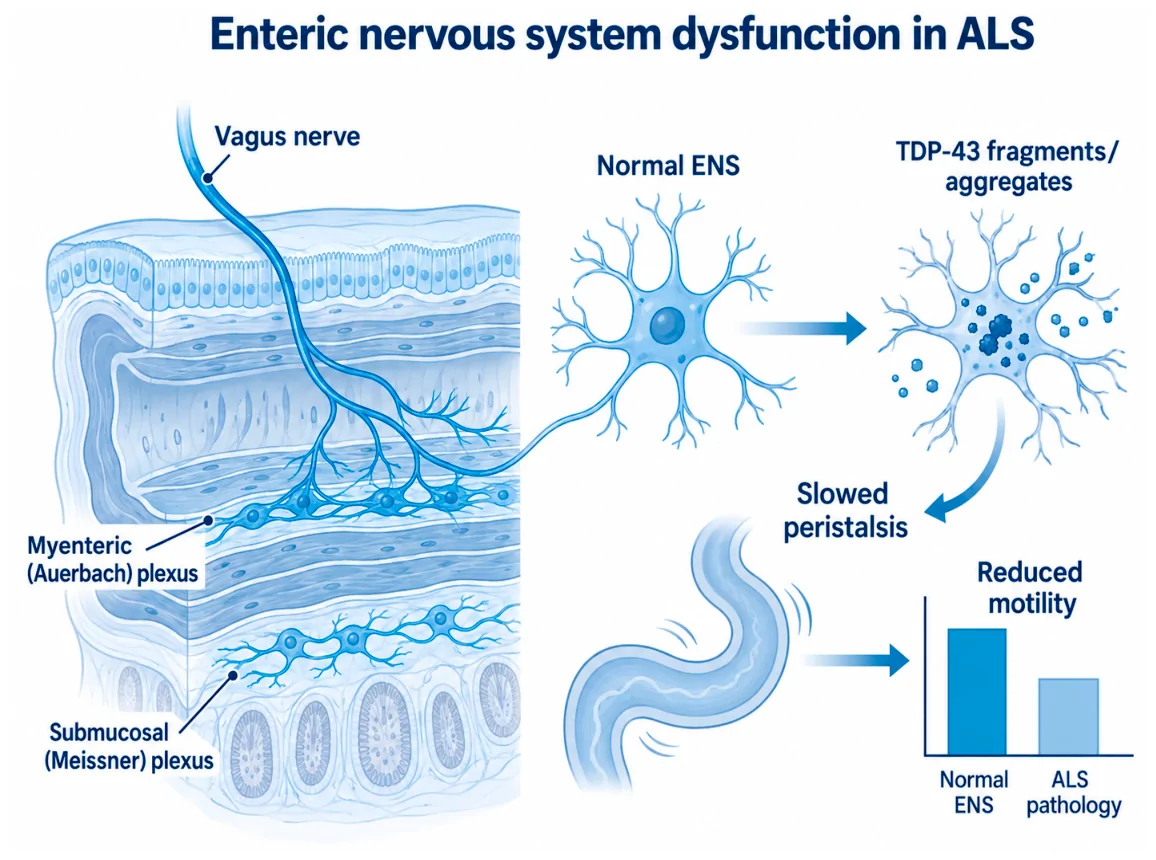
Schematic illustration of potential enteric nervous system involvement in amyotrophic lateral sclerosis (ALS). Experimental TDP-43 models support intestinal dysfunction and abnormalities involving the myenteric (Auerbach) plexus; however, the clinical significance of enteric nervous system pathology in human ALS remains uncertain. ALS, amyotrophic lateral sclerosis; ENS, enteric nervous system; TDP-43, TAR DNA-binding protein 43. The vagus nerve is depicted schematically and should not be interpreted as a demonstrated gut-to-brain propagation route in human ALS. Illustration generated using artificial intelligence for visualization purposes.

**Figure 7:**
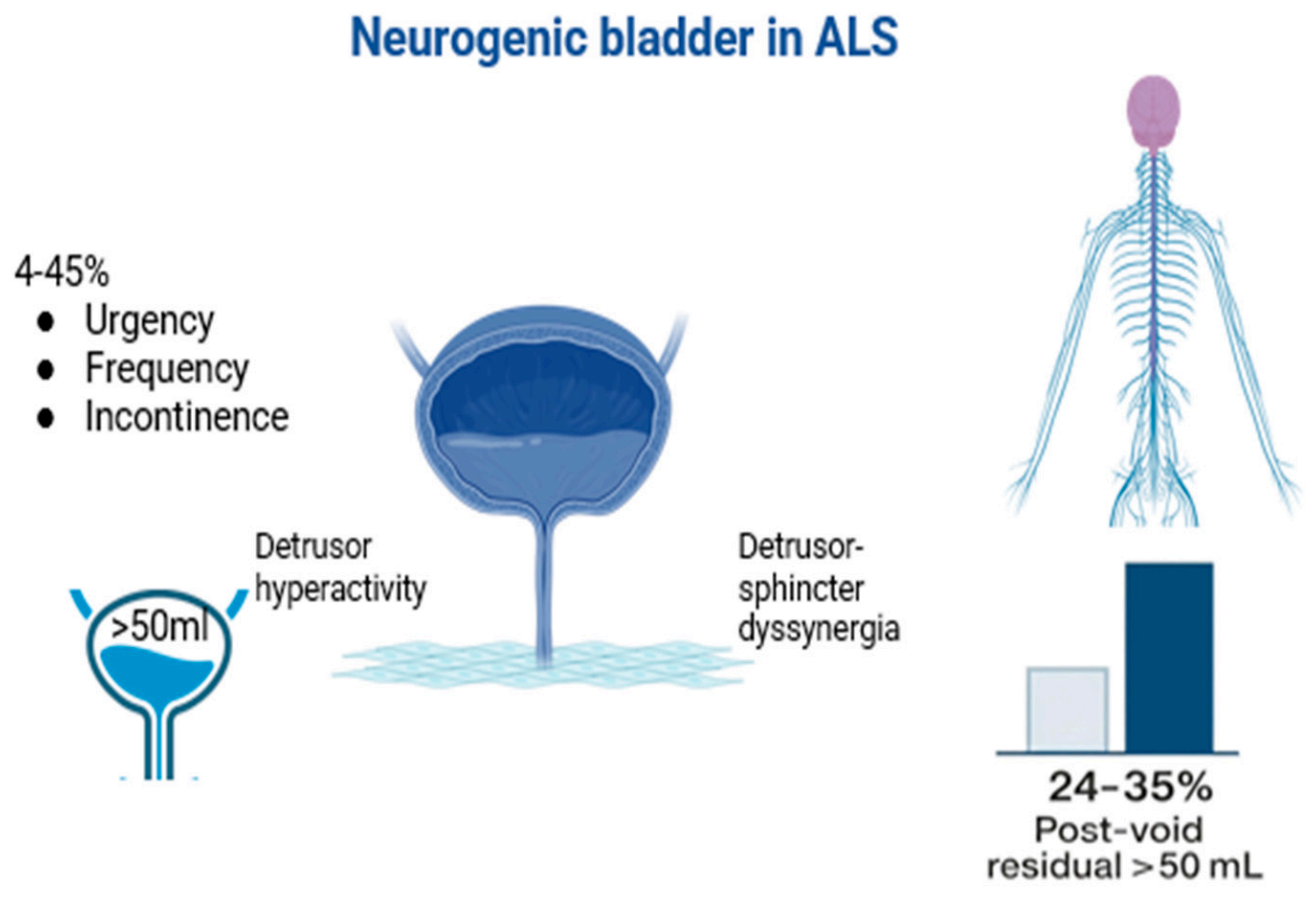
Neurogenic bladder dysfunction in amyotrophic lateral sclerosis (ALS). Lower urinary tract symptoms may include urgency, frequency, and urinary incontinence, while urodynamic findings may demonstrate detrusor overactivity and detrusor-sphincter dyssynergia. Increased post-void residual volume (>50 mL) may indicate incomplete bladder emptying. ALS, amyotrophic lateral sclerosis.

## Data Availability

No new data were created or analyzed in this study. Data sharing is not applicable to this article.
